# A technical semi-field methodology to measure the effect of nutrition on honey bee brood rearing

**DOI:** 10.1242/jeb.251151

**Published:** 2026-01-02

**Authors:** Rui F. S. Gonçalves, Raquel T. de Sousa, Daniel Stabler, David M. S. Pinto, Geraldine A. Wright, Sharoni Shafir

**Affiliations:** ^1^ John Krebs Field Station, Department of Biology, University of Oxford, Oxford, OX2 8QJ, UK; ^2^ B. Triwaks Bee Research Center, Department of Entomology, Institute of Environmental Sciences, The Robert H. Smith Faculty of Agriculture, Food and Environment, The Hebrew University of Jerusalem, Rehovot 76100, Israel; ^3^ School of Biological Sciences, Faculty of Environmental and Life Sciences, University of Southampton, Southampton, SO17 1BJ, UK; ^4^ Visual Geometry Group, Department of Engineering Science, University of Oxford, Oxford, OX1 3PJ, UK

**Keywords:** Honey bee nutrition, Brood production, Semi-field method, Mini-colony bioassay, Protein supplementation, Colony health

## Abstract

A honey bee colony's well-being is its ability to nurture larvae into healthy adults. Understanding how nutrition supports brood rearing is crucial for developing diets that could aid against environmental threats. Nutritional research on whole-colony brood development has been historically challenging because of difficulties documenting the diet's impact on brood production over time. We describe a novel semi-field method to study the influence of nutrition on brood rearing using standardised small colonies formed *de novo* (ca*.* 1500 nurse-age bees and a queen) housed in adapted mating-nucs, placed inside an enclosure and limited to feeding on chemically defined diets. Complete assessments were conducted every 15 days, assisted by a bespoke device to photograph every frame to measure cell contents. A novel metric describes the number of bees generated per gram of diet consumed, measuring the impact of nutrition on brood rearing and overall colony size.

## Teaser

**Summary:** A novel semi-field method standardises small honey bee colonies to quantify how diet composition influences brood rearing through a new bees-per-gram nutritional metric.

## INTRODUCTION

The honey bee colony's capacity to produce brood and generate healthy young workers is a hallmark of robust colony health (vanEngelsdorp et al., 2009; Straub et al., 2015; Mattila and Seeley, 2007). The reproduction of honey bees exhibits a haplodiploid eusocial structure where adult individuals (male, reproductive female and unreproductive females) play distinctive roles in colony survival (Kennedy and Radford, 2020; Joshi and Wiens, 2023). Beyond the queen's polyandrous mating, the population's survival relies on the workers’ ability to maintain larvae, collect food, thermoregulate and fight off pathogens and pests through their specific division of labour (Wild et al., 2021; Page and Robinson, 1991). Foragers collect pollen and nectar from flowering plants, which are processed by nest bees and stored as bee bread and honey, respectively (DeGrandi-Hoffman et al., 2021; Johnson, 2003; Gordon, 1996). Nurse bees with engorged hypopharyngeal glands, between 3 and 12 days old, metabolise food to feed jelly to larvae and the queen (Johnson, 2003; Crailsheim, 1992; Wang et al., 2016; Johnson and Frost, 2012). And colony workers regulate the colony's temperature around 35°C for proper brood development (Jones et al., 2004; Stabentheiner et al., 2010). These tasks require adequate nutrition and precise coordination between nestmates (Johnson, 2010). However, anthropogenic drivers have altered the diversity and abundance of flowering plants in their foraging landscapes, thereby affecting the health of honey bee colonies (Steinhauer et al., 2018; Potts et al., 2010; Dolezal and Toth, 2018; Klein et al., 2017). Starvation conditions are becoming increasingly common, motivating beekeepers and scientists to develop feeds that mimic the composition of floral pollen to feed bees (Brodschneider and Crailsheim, 2010; Ricigliano et al., 2022; Robinson and Nation, 1966). Providing sucrose syrup in the absence of nectar flow, along with protein patties to promote brood rearing (Brodschneider and Crailsheim, 2010; Noordyke et al., 2021), has raised beekeepers’ operational costs related to feeds from 1% in 1976 to 20% in 2018 (Sumner and Champetier, 2018).

Research on colony nutrition has been ongoing since the 1930s (Haydak and Tanquary, 1943), covering a variety of methods in settings such as open fields (Ricigliano et al., 2018, 2022; DeGrandi-Hoffman et al., 2016; Harbo, 1986; Oomen et al., 1992; Mattila and Otis, 2006; Sagili and Breece, 2012; Mortensen et al., 2018; Noordyke et al., 2022), semi-fields (DeGrandi-Hoffman et al., 2021; Robinson and Nation, 1966; Haydak and Tanquary, 1943; Herbert et al., 1970; Herbert and Shimanuki, 1978a; Standifer et al., 1973; Campana and Moeller, 1977; Bakker and Calis, 2003; Höcherl et al., 2012), labs (Arien et al., 2020; Fine et al., 2018; Mortensen and Ellis, 2018) and complex indoor bee-flight rooms (Czoppelt et al., 1980; Pernal and Currie, 2001; Loper and Berdel, 1980). Among the three primary macronutrients (carbohydrates, fats and proteins), proteins play a crucial role in promoting brood rearing (Brodschneider and Crailsheim, 2010), and pollen, the most studied protein source for honey bee colonies, has a substantial positive impact (Keller et al., 2005). However, certain pollen types, such as dandelion and maize (Herbert et al., 1970; Höcherl et al., 2012), are insufficient to support colony growth, and a diverse pollen source is widely regarded as essential for maintaining healthy bee populations (Frias et al., 2016; Pasquale et al., 2016). When protein sources such as pollen are restricted, brood-rearing ceases (Mattila and Otis, 2007), and if carbohydrate stores, such as honey, are absent, the colony enters a period of starvation, putting its survival at stake (Dolezal and Toth, 2018; Brodschneider and Crailsheim, 2010; Schulz et al., 2002; Scofield and Mattila, 2015; Branchiccela et al., 2019). Without pollen, colonies cannot raise brood.

The unreliability of pollen access has led to the development of diets rich in protein and other essential nutrients, which are often absent from natural pollen – referred to as pollen substitutes. However, these still do not appear to demonstrate a clear benefit to colony health (Mortensen et al., 2018). Pollen substitutes are less effective than those diets containing at least 10% natural pollen in sustaining colony populations over the long term (Ricigliano et al., 2022; Mortensen et al., 2018; Lamontagne-Drolet et al., 2019; Wright et al., 2018). Furthermore, when feeding on these diets under conditions of pollen scarcity, bees deplete their internal reserves to support larval development, as they often lack essential nutrients, such as sterols (Brodschneider and Crailsheim, 2010; Noordyke et al., 2021; Scofield and Mattila, 2015; Powell et al., 2023). It remains unclear how long a pollen-free diet can support healthy populations throughout their annual cycle.

In 22 field studies on pollen substitutes, only five showed increased brood production (Noordyke and Ellis, 2021). While valuable for understanding real-world conditions, field studies come with significant challenges. They are prone to uncontrollable variables (environmental, technical or genetic) that affect brood rearing more than feeding protein diets (Ricigliano et al., 2018; Peirson et al., 2024). For example, access to flowering plants and their pollen, even in small quantities, creates a confounding factor in brood rearing, which masks the nutritional effects of the tested diets (Lamontagne-Drolet et al., 2019). Methods to restrict access to foraging pollen, such as pollen traps (Mortensen et al., 2018), harm colony health by damaging workers (Dubois et al., 2018). Hive location may also expose them to plant protection chemicals, affecting colony development (Fisher et al., 2021; Souza et al., 2024). Likewise, using large colonies can lead to the accumulation of other toxins (Morales et al., 2020) used to control *Varroa* mites, affecting brood capping rates, larval survival and bee mortality (Castle et al., 2022; Barascou et al., 2021). They are also more challenging to control for comb age; new combs have larger brood areas and heavier worker bees than older ones (Berry and Delaplane, 2001). Even the spatial distribution of bee bread within the comb affects brood-rearing success (Wang et al., 2020).

In semi-field studies, pollen access and foraging are effectively controlled. However, most use fewer than three colony replicates and rarely track broods over time. Colonies are typically confined in small outdoor cages (<70 m^3^) (Sonter et al., 2019; Corby-Harris et al., 2021), which increases mortality and reduces brood rearing (Herbert and Shimanuki, 1978a). Colony sizes vary from 2000 (Höcherl et al., 2012) to 10,000 bees (Corby-Harris et al., 2021) and the variety of methods for brood counting, such as visual estimation (Delaplane et al., 2013), sampling a central area (Ko et al., 2017) or image analysis tools (Alves et al., 2020), offers different levels of precision in estimating the diet's impact on brood rearing.

Most importantly, comparing the nutritional impacts on brood rearing between studies is challenging without standardising methods and standard measurable metrics. In contrast, standard metrics are employed in honey bee toxicology to evaluate brood rearing. For example, the Oomen bee brood feeding test (Oomen et al., 1992) and the OECD75 guideline (OECD, 2014) are widely used by industry and academic groups that test diets laced with toxicants, measuring the impact of pesticides on brood – via the brood termination rate metric (BTR), which measures the failure of eggs to transition into adults (OECD, 2014). An incidental outcome of these studies was the recommendation to use large polytunnels (>100 m^3^) to mitigate the harmful ‘caging effect’ on colonies (Wang et al., 2020; Szczesniak et al., 2012; Lückmann and Schmitzer, 2019; Becker and Lückmann, 2017). While BTR focuses on brood toxicity, comparable standards are needed to measure the positive impact in diet brood-rearing studies.

Therefore, we developed a technical semi-field method to address this need for standardisation and introduced a key metric, ‘bees per gram’, which measures the number of bees produced per gram of diet consumed. This metric enables the comparison of the brood-rearing potential of different diets across studies, locations and time, in free-flying colonies under completely pollen-restricted conditions. Our experimental set-up used mini-colonies with 1500 mixed nurse bees, five mini-frames with combs built the same year (without bee bread), and a same-year mated queen. Each mini-colony was housed in repurposed polystyrene mini-nucleus boxes (mini-nucs), with 0.002 m^3^ of nest volume and a feeding chamber, and enclosed within a large flight arena – a polytunnel with 628 m^3^ of flight space, or within a 125 m^3^ glasshouse. Sucrose solution feeders were dispersed throughout to simulate natural nectar foraging behaviour. At the 15 day experimental mark, the brood assessment was carried out utilising a custom device to photograph frames and analyse cell contents (empty, honey or brood). This approach minimised confounding factors (e.g. pollen access, colony size, queen or comb age, foraging and agrochemicals), allowing for a precise evaluation of diet effects on brood rearing.

In the present study, we developed an optimised brood assessment framework, showcasing key techniques that minimise disturbance to the colony while providing precise data on brood development. Our practical, reproducible approach streamlines the transition from laboratory nutritional studies to practical applications that can directly benefit beekeepers. We hope it serves as a foundation for standardising future whole-colony nutrition research.

## MATERIALS AND METHODS

### Nutritional bioassay

**Fig. 1.**
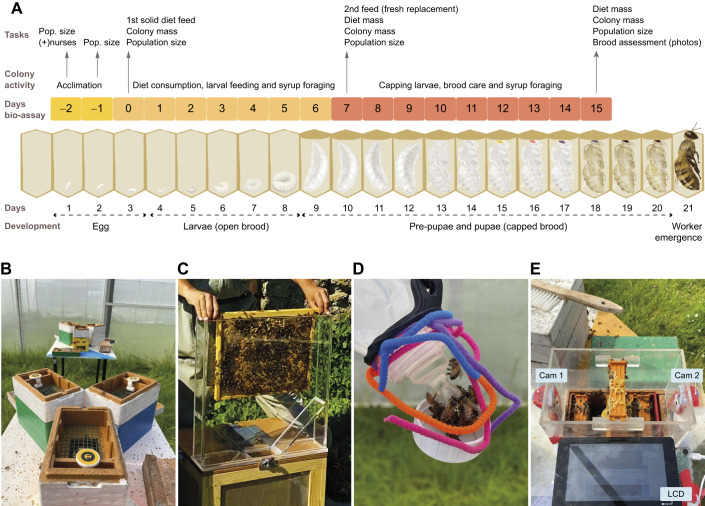
**The brood assessment bioassay.** (A) The chart matrix displays the honey bee development stage each day, stacked with bioassay days. Negative days represent the acclimation period, and days 0–15 represent the feeding period, which occurs within a 15 day cycle, followed by a final brood assessment. From left to right, the main tasks are as follows: on day 0, assess colony mass, population size and feeding each with a solid diet (patty); on day 7, colony mass, population size and the mass change in the patty are used to estimate diet consumption; on day 15 (one brood cycle), assess the same metrics and conduct a full brood observation, by photographing every frame across all treatments for a capped brood count. The chart also describes the mini-colony's main activity: after acclimation inside the semi-field enclosure, it progressively consumes the diet to feed the larva, forages for nectar syrup and ultimately takes care of its brood. All treatments started at the same brood stage – egg. (B) Mini-colonies in groups of three nucs in a triangle-shaped configuration; the picture displays the solid food chamber with the mesh supporting diet and a temperature sensor. (C) Nurse bees are collected and placed into the ‘nurse collection box’ to replenish each mini-colony before the experiment begins. (D) Honey bees forage on artificial flowers with attached coloured velvet wires and bottle caps for support. (E) A novel, custom-made device built from acrylic sheets comprising a box stand to support a Raspberry Pi LCD, with two cameras, and displaying a bee frame ready for photography.

Our nutritional bioassay is based on queen-right buckfast honey bee (*Apis mellifera* Linnaeus 1758) colonies exclusively consuming artificial diets, which are then metabolised by nurse bees to feed their worker larvae. The developmental cycle of workers, from egg until emergence, usually takes about 21 days. Our assessments track this cycle almost to its end, from egg until capped pupae – 3 days before emergence (Fig. 1A).

Our method comprises three parts: acclimation, feeding and brood assessment. An initial set-up phase, represented in Fig. S3, is required to first establish the mini-colonies inside the controlled flight enclosures. This initial set-up takes 5 days, where day 0 is the beginning of feeding. The feeding cycles repeat from days 0 to 15. Consumption and the bee population are monitored during the 15 day cycle, and a full brood assessment is conducted at the end. If, in between the 15 day feeding cycles, the mini-colony consumes the diet and has more than two frames of bees, the system progresses through the feeding cycle uninterrupted. However, if the bee population criterion is unmet, it triggers a nurse addition stage and colony acclimation. This is an additional 2 day process that involves adding nurse bees to low-populated mini-colonies, and the system resumes the standard 15 day cycle. This dynamic ensures that the system remains adaptive and maintains a colony baseline throughout its cycle. The method operates in a continuous loop with a repeating 15 day feeding cycle; however, we conducted only one cycle in this study.

### Part I – laboratory diet design

Our chemically defined diets consisted of protein, carbohydrates, fats, sterols, micronutrients and a maltodextrin filler (with no nutritional value for the bee). We used as a protein source a plant-based product – Soy Protein Isolate (Myprotein, Northwich, UK; 90% purity) – as described in Sereia et al. (2013). Here, we selected six diet treatments varying only in protein content (P, percentage w/w): 6% (P6), 12% (P12), 15% (P15), 18% (P18), 25% (P25) and 30% (P30). The percentage (w/w) of the diet total carbohydrate mixture ranged from 38% for P30 to 68% for P6, depending on the diet treatment. The carbohydrates consisted of about two-thirds of a mixture of 80% w/w sugar solution (in an equal mix of glucose and fructose) and one-third sucrose powder (Kent Foods, Glasgow, UK). As the fat source, we used soy lecithin (Optima Lecithin Health & Nutrition) at 6% w/w of the total diet on all treatments as in Stabler et al. (2021). We used cholesterol (Sigma-Aldrich, ≥99%) at 0.1% w/w of the total diet as the sterol source, adapted from Herbert and Svoboda's (1980) diets (Svoboda et al., 1980; Herbert et al., 1980). We then mixed all ingredients into a dough and kneaded them into 50–60 g (5 mm thick) patties. To test the pliability and texture of the dough formulations (structure tests), we further tested them by placing samples on top of a plastic mesh (1 cm diameter) inside an incubator at 34°C and 60% humidity (simulating in-hive conditions). After 24 h, we discarded the diet formulations that absorbed excessive moisture and turned into sludge. We finally selected diets that successfully passed the structure test and that had been previously tested in our lab for palatability, consumption and bee survival (>90%) in ventilated acrylic boxes (authors' unpublished data), monitored over a week as in de Sousa et al. (2022).

### Part II – mini-colony preparation and set-up

We assembled 36 mini-nucleus (mini-nuc) Styrofoam boxes with dimensions 24×15×16.5 cm (Apidea Vertriebs AG, Zug, Switzerland), fitted with three plastic mini-frames with an 11×11 cm new wax foundation and one internal liquid feeder with clay pebbles inside to prevent bees from drowning. Each mini-nuc was number tagged and painted in separate colours to facilitate homing behaviour (Capaldi and Dyer, 1999). To measure enclosed temperature fluctuations, we added a temperature sensor data logger (Omega, OM-EL-USB-1) to the food chamber of a small subset of mini-colonies.

To minimise the influence of pathogens and viral diseases on our experimental nurse bees, our apiary colonies had been treated with oxalic acid and amitraz between February and April, and *Varroa* levels were monitored using the sticky board method. We then selected healthy colonies from our apiary that were free of visible disease symptoms (i.e. the presence of deformed wing virus, DWV) and with no detectable *Varroa* infestation. On day −5 we started collecting a selection of mixed nurse bees from these colonies by gently shaking open-brood frames covered with nurse bees into our custom-made wooden nurse collection box (37.4×42.2×31.4 cm) (Fig. 1C). This box, covered by insect metal mesh, had three internal frames, spaced at 6 cm one from the other, allowing bees to suspend in cluster formation to dissipate heat in the field. A detachable acrylic funnel was positioned on top, facilitating the brushing of nurse bees into it. Nurse bees were brushed into the box while lightly sprinkling them with a 33% sugar solution laced with a couple of drops of lemongrass oil (Neal's Yard, Covent Garden, London) to prevent them from flying and to encourage nestmate formation (Slessor et al., 2005). Nurse bees must be brushed at a rate of 1.5 frames of open brood per mini-nuc, scaled to the total number of mini-nucs used (e.g. brushing 15 frames for 10 mini-nucs). Once this was completed, the box was allowed to rest for 1–3 h inside a dark room at 10°C to promote cluster formation. At the time of nurse collection, no *Varroa* mites were detected, and no signs of DWV or other disease symptoms were observed. As colonies showing visible signs of pests or diseases are behaviourally compromised and may influence brood rearing, any bees or colonies carrying mites or exhibiting visible signs of DWV were excluded.

Next, we turned each mini-nuc bottom up, sliding open the floor panel and exposing the three plastic mini-frames with the foundation. Lining all mini-nucs (apideas) in a row, we sequentially placed on each a caged mated queen with candy (Candito per Api, PIDA, Albignasego, Italy) between the 2nd and 3rd frames. Our mated queens were sister queens supplied by the same Buckfast breeder. Next we used a 500 ml glass beaker to scoop the bees off the collection box frames, filled each mini-nuc with 450 ml of mixed nurse bees (approximately 1018) inside, and then slid closed the floor panel. Once all mini-colonies were formed *de novo* with nurse bees and a mated queen, we fed 350 ml of 1:1 (water to sugar) syrup into the internal feeder. We placed all mini-colonies housed in mini-nucs inside a ventilated dark room at 10°C for 48 h for further nestmate and colony formation.

### Part III – mini-colony acclimatisation to semi-field enclosures

We conducted two experiments, the first in a glasshouse with 30 colonies and the second in a polytunnel with 6 colonies. For the first experiment, after the 48 h of nestmate formation (day −3), at sunset, we released 30 mini-colonies inside the two-room glasshouse (10×5×2.5 m) with 125 m^3^ of total flight volume to acclimate to the semi-field setting and to test our five increasing stepwise protein diets (P6, P12, P18, P25, P30). The repurposed glasshouse consisted of glass panels with a galvanised steel frame structure. Each of the two rooms of the glasshouse had a long central table where we placed 15 mini-colonies in groups of three (triangle shape) with entrances pointing in different directions. Because of the inherent heating capacity of the glasshouse infrastructure and to promote air circulation, we installed a mesh shade covering the whole area of the glass ceiling, two commercial extraction fans (one on each end of the glasshouse), three portable aircon units and three oscillating pedestal fans.

The second experiment was conducted in a purpose-built polytunnel (NP Structures, Colne, UK) measuring 18.4×8.23×4.15 m, providing 6 times more flight volume (628.4 m^3^) than in the glasshouse. The polytunnel was covered in white insect mesh, and we repeated the same method with the P15 diet using 6 new mini-colonies, which were placed in different directions in two groups of three, each on two tables, as described above.

Twenty-four hours after releasing the mini-colonies inside the semi-field enclosures (day −2, see Fig. 1A), we expanded their nest volume by removing the internal liquid feeder, adding two frames with empty built-in wax comb, and a top solid food chamber. During this procedure, we checked for queen release and visually confirmed that all queens had been naturally released, after which all empty queen cages were removed. If, for instance, queens remained caged, we manually removed the candy plug and reinserted the cage to allow a slower, controlled release. Mated queens usually start laying eggs 24 h after their release. Additionally, we kept in stock empty frames with built-in combs to standardise for frame composition, replacing any of the initial three foundation frames that were not yet fully drawn. We then topped up with an extra 250 ml of mixed nurse bees (approximately 566 bees) from our nurse collection box (Fig. 1C). The solid food chamber, used for topping-up nurse bees and placing the protein diets, was made from an Apidea Feeder Super (700 ml), into which we cut a central hole (10×8 cm) and covered it with a green plastic mesh (12.5×9.5 cm, diameter 1 cm) (Fig. 1B). At this stage, a total of 700 ml of mixed-aged bees (corresponding to approximately 200 g and around 1580 bees) were added to each colony, close to 5.2 bee seams (clusters of bees occupying the spaces between two combs, or comb and hive wall). Each bee seam was estimated to have approximately 300 bees. This estimation is based on an average of 787 bees per 100 g and 226 bees per 100 ml, calculated from 28 research papers, including our own measurements.

Next, we suspended two artificial flowers per mini-colony throughout the enclosures, made from adapted 250 ml infant feeding bottles by piercing five 0.5 mm diameter holes in the nipples and adding colourful velvet pipe cleaners for bee identification and grip (Fig. 1D). Each bottle was filled with 250 ml of 33% sucrose solution and hung upside down, functioning as a gravity feeder to promote nectar foraging. Our artificial flowers restricted bees’ drinking access to only five at a time, preventing hoarding behaviour. The sucrose concentration in our feeders is in the mid-range of nectar of flowers typically visited by honey bees (Pamminger et al., 2019), and extends foraging activity within the enclosures relative to using higher sucrose concentrations. Additionally, such syrup concentration maintains high internal humidity favourable to brood development in summer and engages nest bees in activities of nectar-to-honey conversion. Drawbacks of low syrup concentration were noted during periods of continuous rain and cold within the polytunnel, leading to an outbreak of chalkbrood, consistent with findings by Flores et al. (1996). We washed and replenished feeding bottles every week and as needed. If, for instance, bees hoarded syrup, completely filling most of the comb cells in the mini-nucs, and thereby obstructing egg laying, we replaced those frames during our inspection days with our in-house stock of empty built combs. We also placed four tubs with fresh water and support rocks in each corner of the enclosures for water supply.

Our mini-colonies were considered ready when they consisted of a single five-frame brood chamber with the additional top food chamber (Fig. 1B). Internally, colonies contained five frames of fully built combs populated with bees (approximately 1500 individuals), sugar syrup reserves and brood exclusively at the egg stage produced by a mated queen.

### Part IV – mini-colony nutritional bioassay for brood assessment, colony mass and population size estimation

We assigned *n=*6 mini-colonies to one of each of five diet treatments (P6, P12, P18, P25 and P30) in the glasshouse enclosure and to P15 in the polytunnel enclosure. The assignment criteria of diet to mini-colony aimed to maintain a balanced population size distribution and entrance direction among treatments and reduce bias on sun exposure, orientation and colony population. Before feeding the mini-colonies with the protein diets, we recorded the initial mass of each mini-colony with a field scale (Ohaus Scout Pro Portable). We also recorded the number of bee seams (0–6 bee seams) as per Chabert et al. (2021) by photographing the colony from above to estimate the population size of each mini-colony. Each bee seam was estimated to represent approximately 300 bees to calculate the colony size.

For all treatments, we weighed each diet onto 50–60 g of dough, rolled them into 5 mm thick patties, and placed them inside the food chamber over the green mesh, which sits 1 cm above the brood frames. Bees could access the food from below and move within the food chamber (Fig. 1B) to consume the diet. This marks the start of our nutritional bioassay experiment (day 0).

A week later (day 7), with minimal disturbance to the colony, we removed the patty and registered population size and diet consumption by tracking changes in the patty's mass for all mini-colonies and diet treatments (Fig. 1A), taking an observation note if bees were eating the patty. The treatments were discontinued if nurse-aged bees avoided the diet altogether, indicated by marginal changes in diet mass or ‘negative consumption’, i.e. when patties are not eaten and instead gain mass as a result of moisture absorption. In both experiments, the feeding cycle proceeding from consumption was observed in all treatments. Therefore, in our study, a fresh patty was provided, and its mass was recorded before adding the new patty. The mini-colonies were weighed to estimate changes in sugar stores.

On day 15 of our bioassay experiment (one brood cycle), we performed a complete mini-colony assessment on all treatments. We assessed colony mass, population size, honey stores, diet consumption and brood stages on all frames (Fig. 1A). We measured the brood stages by photographing both sides of every frame using a bespoke photographic-acrylic device (Fig. S1). The device consisted of a custom-made acrylic box (internal dimensions: 22.3×14.5×13.2 cm, 4 mm thickness) that could be mounted on top of an open mini-colony rim, with a one-frame stand at its centre. It had two RPi cameras (64MP Autofocus Camera, Arducam), one on each end, focused on the frame's centre, with both cameras connected to a Raspberry Pi 4 (designated as the imager). Another Raspberry Pi 3 (named the controller) was connected to a touchscreen display (Official Raspberry Pi 7″ Touchscreen Display) and to the imager via an Ethernet cable (Fig. 1E). The controller controlled the imager remotely via SSH using the shell script software ‘image-experiment.sh’. Detailed set-up instructions and the code used are available from GitHub (github.com/carandraug/beehive-imaging) under the GNU All-Permissive License. The device recorded the time taken for the complete assessment (five frames, 10 pictures per mini-colony serially numbered); an experienced researcher spent, on average, 5 min and 41 s per mini-colony from setting the device to removing it. We then visually counted the number of capped brood cells in each image using available software (Adobe Creative Cloud 2019-2024, version 6.4.0.361, PS -Count Tool). At this stage of our experiment, we measured the influence of nutrition during the ‘one-capped bioassay cycle’ (Fig. 1A). We defined each bioassay cycle as the period when all capped brood originated from eggs that were laid under the experimental procedure. At the start of the feeding period, all treatments started at the same brood stage (eggs) at day 0, with no capped cells present.

Not all of the eggs that the queen lays will develop into adults. Nutritional stress may lead to oophagy, the consumption of eggs by nurse bees, and to cannibalistic consumption of the young larvae (Brodschneider and Crailsheim, 2010). Developing brood that is sick may be identified by nurse bees, which will open capped cells and remove the sick bee (Leclercq et al., 2017). However, a healthy brood that reaches the capped stage will probably emerge as an adult. In the subsequent 15 day bioassay cycles, the previously capped brood will emerge, the newly laid eggs will turn into pupae and a new cycle will begin, guaranteeing that we are not double-counting the same capped cells or missing capped cycles. Hence, we can continue the experiment for additional bioassay cycles by sequentially repeating the procedures from day 7 and day 15, measuring each newly capped cell that is in the stage between the pre-pupae and the point before the pupae emerge. In this study, we terminated the experiment after the first bioassay cycle.

### Data, statistics and power analysis

Statistical analyses of diet consumption, capped-brood-cell counts, protein consumption and ‘bees per gram’ were conducted in Python 3.12.4 (www.python.org) within VS Code, with assistance from GitHub Copilot. Models and related functions from Statsmodels 0.14.1 (www.statsmodels.org) with numpy 2.2.3 and pandas 2.2.3 were used for all tests. We removed three mini-colony replicates from the analyses that lost their queen and had zero capped brood at the end of the 15 day bioassay.

To analyse diet consumption during the nutritional bioassay, we measured the total diet consumed over the 15 day period. Protein ingestion was estimated by multiplying each diet's protein percentage by its total consumption. We then calculated our metric ‘bees per gram’ by dividing the number of capped brood cells by the total diet consumed. This metric represents the number of bees reared per gram of diet that is consumed, based on the assumption that each capped cell will develop into a viable adult bee. A Pearson correlation matrix was used to assess relationships between the independent variable (diet type) and dependent variables, including capped brood, total diet consumption, protein ingestion, population size and ‘bees per gram’.

Our main objective was to evaluate the effects of nutrition, specifically protein content, on brood development. We explored the relationship between protein percentage as a continuous variable with our response variables – consumption, capped brood cells and ‘bees per gram’ – by using the ordinary least squares (OLS) regression model from the Statsmodels 0.14.1 package. We estimated the intercept of the regression line to make it more accurate for our biological data. Key outputs include: the coefficient (coef), which indicates the strength and direction of the protein influence; the *P*-value and the *R*^2^ value, which quantifies how much of the variation in the response is explained by protein. We also used the OLS to perform a power analysis to estimate: (i) the probability of accurately detecting the protein effect on the brood−power (1−β) and (ii) considering that in future studies, we require an 80% degree of confidence, what is the sample size needed. We used Cohen's *f*, √[*r*^2^/(1−*r*^2^)], to calculate the effect size and set the alpha level at 0.05.

The relationship between bee population size and capped brood was analysed by averaging population size between the three time points assessed during the feeding period: day 0, day 7 and day 15. Population trends were analysed using Graphpad prism 10.4.1. To analyse population trends throughout the days and population differences between diets, we employed its mixed-effects model with restricted maximum likelihood (REML) estimation at a significance level of 0.05, assuming that the variance between treatments was equal. Specifically, the mixed-effects model evaluated population differences between diet treatments inside the glasshouse (P6, P12, P18, P25, P30) on each assessment day and assessed population changes over time for each diet. This analysis covered the feeding period (days 0, 7 and 15) and the acclimation period (days −2 and −1). We then used Tukey's *post hoc* multiple comparison test to determine population differences between days and treatments. As population size in mini-colonies can be a confounding factor for rearing capped brood, and as we observed absconding events among mini-colonies inside the glasshouse, we set criteria to identify potential outliers. Specifically, if a mini-colony showed a significant difference in population size in our mixed-effects model between day 0 and day 15, and its capped brood measurement had a *z*-score exceeding the factor of 2, meaning a data point beyond two standard deviations from the mean, we considered that measurement an outlier. The *z*-score (*z*) for each data point (*x*) was calculated using the formula: *z*=(*x*−μ)/σ, where μ is the mean of the data and σ is the standard deviation. Those outliers were not accounted for in statistics but are represented in figures.

To analyse the relationship between the two enclosures, polytunnel versus glasshouse, we selected a subset of our glasshouse data, the P18 diet, to compare it with the diet that was tested inside the polytunnel, the P15 diet. Here, we used a simple *t*-test to compare these two groups’ means and establish whether the difference between them was statistically significant. In this case, we used Graphpad Prism 10.4.1 *t*-test model.

## RESULTS AND DISCUSSION

### Protein-rich diets increase both diet consumption and protein intake

**Fig. 2.**
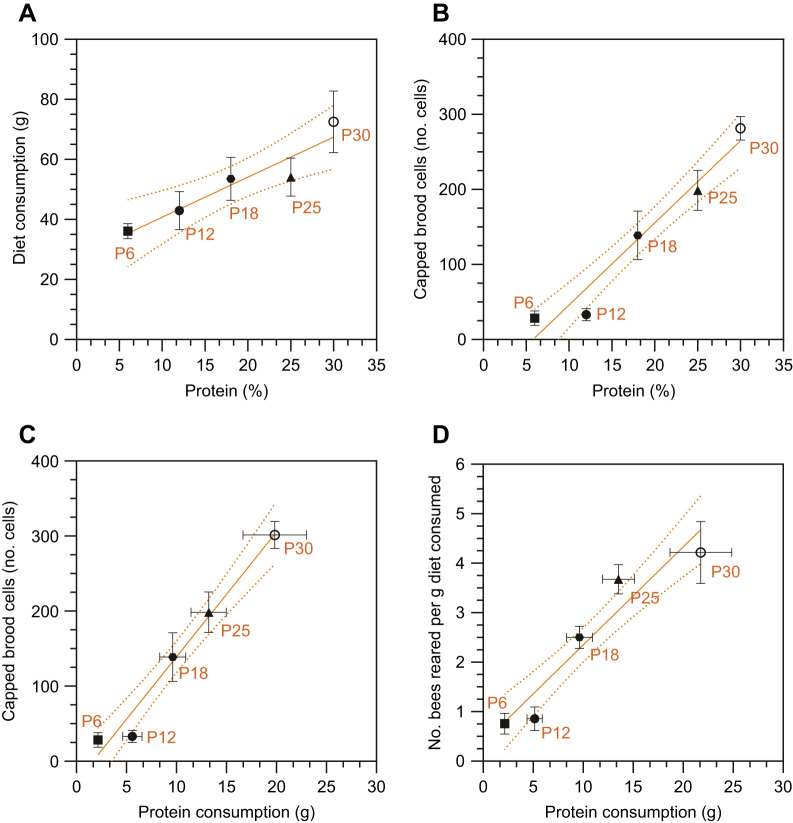
**Protein content proportionally influences diet consumption, capped-brood rates and the number of bees reared in mini-colonies.** Data points in all figures show the mean results for each of the five diets varying in protein content (% w/w) inside the glasshouse: P6 (6%), P12 (12%), P18 (18%), P25 (25%) and P30 (30%), over the 15 day period (one bioassay cycle). Orange lines depict the regression lines with 95% degree confidence between different diet types. Error bars are s.e.m., *n*=5 mini-colonies per P6, P12 and P30 diet; and *n*=6 mini-colonies per P18 and P25 diet. (A) The relationship between dietary protein percentage and total diet consumption over the 15 day period. A positive linear association was found, with the ordinary least squares (OLS) model showing that higher protein content is associated with greater consumption. (B) The relationship between dietary protein percentage and the total number of capped brood cells counted at the end of the 15 day period. A positive association was found, showing that higher protein content significantly predicted more capped cells. (C) The relationship between protein intake and the number of capped-brood cells shows a positive association, with protein-rich diets leading to higher protein intake and a higher number of capped cells. Our linear regression shows that for every gram of protein ingested, mini-colonies can generate 12 more bees. (D) The relationship between protein intake and our metric ‘bees per gram’ (number of bees reared per gram of diet consumed). Mini-colonies on protein-rich diets (e.g. P30) were, on average, able to rear 4 bees per gram of diet consumed, while diets lower in protein (P6 and P12) produced <1 bee per gram of food consumed.

The mini-colony's access to pollen was entirely restricted and pollen was absent from all comb stores, with protein provided solely through the experimental diets delivered to the mini-nucs throughout the 15 day bioassay cycle. We performed an OLS model to evaluate the influence of protein percentage on diet consumption (Fig. 2A). The OLS showed that protein-rich diets positively affected diet consumption (β=1.33, *P*<0.005), indicating that for each protein percentage increase in the diets, our mini-colonies ate 1.3 g more. The model explained 35% of the variance in diet consumption (*R*^2^=0.35, *F*_1,25_=13.6, *P*=0.001). Additionally, the Pearson correlation demonstrated a positive correlation between protein-rich diets and diet consumption (*r*_25_=0.59, *P*<0.005). Although protein strongly influences diet consumption, it does not necessarily mean colonies will neglect diets that are entirely devoid of protein. For example, other dietary components, such as carbohydrates, can also significantly influence food collection and consumption in honey bee colonies (Paoli et al., 2014; Hendriksma et al., 2019; Bouchebti et al., 2022).

As diet consumption and protein intake were inherently linked in our experiment, mini-colonies that consumed more of the protein-rich diets also ingested more protein (Fig. S1). Here, the OLS model showed a strong fit, explaining 80% of the variance in protein intake (*R*^2^=0.80, *F*_1,25_=104.2, *P*<0.001). The regression coefficient indicated that for every gram of diet consumed, colonies ingested approximately 0.35 g of protein (β=0.35, *P*<0.001). A Pearson correlation further confirmed a strong positive association between diet consumption and protein intake (*r*_25_=0.90, *P*<0.005) (Fig. S1). These results indicate that mini-colonies consumed more and ingested significantly more protein when offered protein-rich diets (Fig. 2A; Fig. S1), suggesting that dietary protein may have both phagostimulatory and metabolic effects that enhance consumption.

### Protein-rich diets strongly influence the number of capped brood cells and produce significantly more bees per gram of diet consumed

Protein plays a critical role in the ability of a honey bee colony to produce healthy young workers, as brood rearing ceases without an adequate protein source. One of our primary objectives was to assess how dietary protein content influences brood production and whether a stepwise increase in protein percentage drives greater protein intake and brood rearing. As shown in Fig. 2B, we observed a clear positive relationship between dietary protein percentage and the number of capped brood cells. The OLS demonstrated a strong fit, explaining 76% of the variance in capped brood production (*R*^2^=0.76, *F*_1,25_=78.61, *P*<0.001). For every 1% increase in dietary protein, colonies produced an average of 11 additional capped cells (β=10.9, *P*<0.001). A power analysis based on our sample size of 27 mini-colonies indicated a 99% probability (power=0.99) of detecting a protein effect within the tested range. If in future studies a power of 80% were acceptable, only five colonies (*N*=5), one per diet level, would be needed to detect a similar effect.

A consistent trend was observed for the relationship between protein intake and brood production (Fig. 2C). The OLS model showed a strong fit, explaining 74% of the variance in capped brood (*R*^2^=0.74, *F*_1,25_=69.56, *P*<0.001). For every gram of protein consumed, mini-colonies produced an average of 12 additional capped brood cells (β=12, *P*<0.001). Protein-rich diets had a marked impact on brood production. Colonies fed the highest-protein diet (P30) produced more than twice as many capped brood cells as those on P18, and nearly 10 times more than colonies on the lowest-protein diet (P6). These findings support our hypothesis that total protein intake – rather than overall diet consumption – is the primary driver of brood development. Diets with higher protein content significantly enhanced brood production, suggesting that increased protein availability improves colony productivity by increasing worker numbers, which may in turn benefit overall colony health.

The ability to precisely quantify both diet consumption and the number of capped brood cells enabled us to estimate diet efficiency, defined by our key metric: bees per gram – the number of bees produced per gram of food consumed (Fig. 2D). The mini-colonies fed diets with low protein content, e.g. P6 and P12, generated less than one bee per gram, whereas protein-rich diets, e.g. P25 and P30, led to 4 times more bees per gram of diet, 3.68±0.29 and 4.21±0.62 bees g^−1^, respectively (means±s.e.m.). This relationship between protein and brood is consistent with previous studies, such as Ricigliano et al. (2022) and others (Mattila and Otis, 2006; Noordyke et al., 2022; Herbert et al., 1977; Herbert and Shimanuki, 1978b, 1982, 1983; van der Steen, 2007), demonstrating a positive relationship between protein supplementation and brood development. Similarly, Li et al. (2012) identified pollen substitutes containing 30–35% protein as the most influential factor for promoting bee development.

To our knowledge, this brood bioassay method is the first to estimate the number of bees a protein diet can produce and evaluate the influence of protein on brood development in a stepwise approach. The novelty lies in demonstrating that a stepwise increase in dietary protein content leads to increased diet consumption (Fig. 2A), and that greater consumption of protein-rich diets results in a compounded intake of total protein (Fig. S1). Furthermore, consumption of protein-rich diets was associated with a higher number of capped brood cells produced (Fig. 2B,C), ultimately improving brood production efficiency, as evidenced by a greater number of bees produced per each gram of diet consumed (Fig. 2D). Future studies should incorporate a positive control using pollen-based diets and a negative control consisting solely of carbohydrates.

### Supplementation of nurse bees and population dynamics in mini-colonies: population is affected by supplementation but not by diet treatment

Mini-colony populations were formed by mixing unrelated nurse bees into a single group, orphaning them by keeping them queenless for 3 h and spraying them with sugar syrup laced with lemongrass (Downs et al., 2000). We measured population sizes during the acclimation period (days −2, −1 and 0) and standardised the colony population before feeding.

**Fig. 3.**
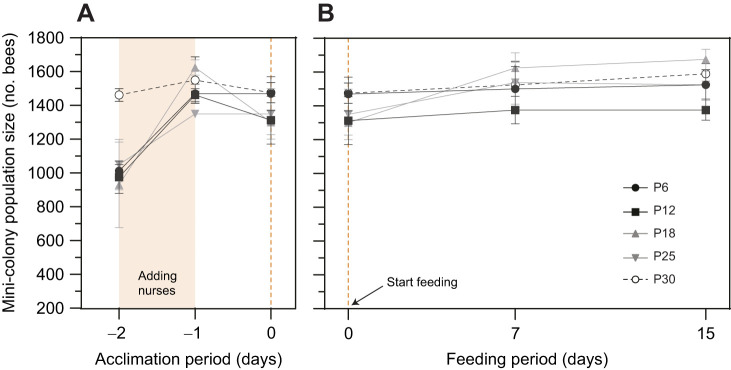
**Population size remains stable during the feeding period.** (A) Population dynamics inside the enclosure (glasshouse) during the acclimation period highlighting the addition of extra orphan nurse bees between day −2 and day −1 (shaded area) (∼500 bees) and the start feeding day 0 (orange dashed line). Nurse addition increased the low populated colonies (mixed-effects model REML, days *P*<0.0001), standardising colony populations between mini-colonies and diet treatments at the start of the protocol (day 0) with similar population sizes of 1400–1500 bees [n.s., mixed-effects model with restricted maximum likelihood (REML), day 0 *P*>0.005]. (B) Population size inside the glasshouse over the 15 day feeding cycle remained stable on most protein diets except P18. There were statistical differences in population trends from day 0 to day 15, on the P18 diet (mixed-effects model with REML, days *P*<0.05, Tukey HSD; *P<*0.005) due to absconding bees randomly entering these colonies. There were no statistical differences between bee populations based on the diet type (n.s., mixed-effects model with REML, diet *P*>0.05). Data are means±s.e.m.

On day −2, at least 24 colonies required supplementation, with fewer than 1500 bees, an average population size of ∼1050±146 bees. One day after supplementation (day −1), the populations increased to 1530±202 bees (Fig. 3A; mixed-effects model, three time points; *P*<0.0001). And on day 0, we observed a slight population reduction, probably due to disorientation and losses within the enclosure. Despite this, supplementation increased the population to a mean of 1422±195 bees on day 0, close to our target (Fig. 3B). This technique effectively standardised colony populations before the feeding period (day 0).

During the feeding period, between day 0 and day 15, we did not anticipate significant differences in population size over such a short time frame unless elevated adult mortality were present. In fact, colony size did not differ significantly between diets (Fig. 3B; mixed-effects model, *P*>0.05), and any influence of colony size on brood development in our semi-field mini-nucs is therefore likely to be minimal. Small colony size might still disrupt honey bee behavioural flexibility, and we cannot exclude the presence of precocious pollen foragers despite protein patties being available to each mini-nuc and natural flowers being absent in our enclosures. Our use of external slow-release artificial nectar flowers, rather than internal syrup feeders, aimed to maintain a functional equilibrium between foragers collecting nectar and housekeeping workers attending the brood.

Colonies were formed in two steps: on colony formation day, outside the enclosure with three mini-frames of bees, and on day −4 (24 h after being released inside the enclosure) by adding two more frames and extra bees to expand the brood chamber. Previously, we attempted to form colonies fully on colony formation day with over 1500 bees and five frames, but faced high mortality as a result of poor ventilation in the mini-nuc and limited syrup consumption during the critical 48 h cold, dark period. To avoid these issues, we adopted the intermediate supplementation step on day −2. Regarding behavioural observations, orphaned nurse bees behaved like natural swarms, forming cohesive clusters with little flight and showing plasticity in nestmate acceptance. The nurse collection box effectively created and replenished mini-colonies. Brief aggression toward non-nestmate nurses was noted in a few colonies but resolved within 2 h.

Assuming that the average lifespan of a worker honey bee is approximately 30 days, worker mortality will be more visible in these mini-nucs. In cases of extended bioassays, the periodic replenishment of mini-nucs, as described in Materials and Methods, will help maintain a stable worker population, and because supplementation and absconding can affect colony nutrition, it is crucial to synchronise these events across treatments and in between the 15 day intervals, while also recording any instances of absconding. The pooling of nurse bees from multiple healthy donor colonies further minimises inter-colony variability in protein and lipid reserves, thereby standardising the overall nutritional status across treatments. Collectively, our results indicated that neither colony demography nor nurse nutritional variation significantly influenced brood rearing.

### Enclosure systems strongly influence ambient temperature and significantly influence brood health

The method described was developed over 5 years, with trials involving different mating-nucs, enclosures, colony sizes and feeder configurations, until it reached the refined stage presented here. Maintaining the daily average ambient temperature inside the enclosures below 37.5°C is crucial, as higher temperatures negatively affect brood survival (Stabentheiner et al., 2010). For example, Xu et al. (2023) showed increased mortality in pre-pupae and pupae at 40°C (Xu et al., 2023).

**Fig. 4.**
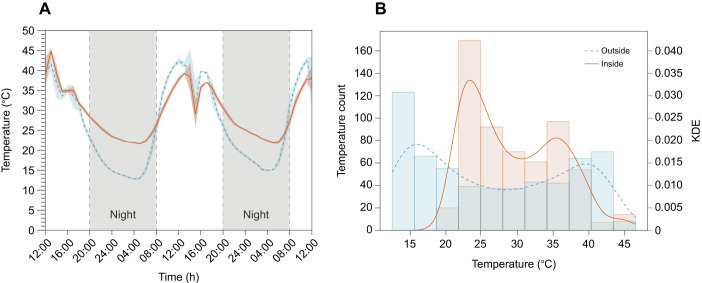
**The mini-colony population can buffer environmental temperature fluctuations.** Temperature fluctuations inside the glasshouse, where orange lines represent fluctuations inside the mini-colony (*n*=2) and blue lines outside the mini-colony (*n*=2), over a 60 h (2.5 days) period during a July heatwave. (A) Maximum environmental temperatures reached 45°C during the daytime, and the minimum temperature was 14°C during the night-time (grey shaded area). The temperature drop at 16:00 h resulted from the action of the misting system, which began at 11:00 h and continued until 20:00 h in 30 min bursts, to cool the environment. (B) Histogram temperature variation between the inside (orange) and outside (blue). The columns represent the temperature histogram count chart (bin width=3), and the lines represent kernel density estimates (KDE), which are probabilities based on the histogram data. The hive's internal temperature was more stable than the outside temperature, with a narrower range and less variability.

In July 2019, the glasshouse ambient readings from the supporting tables (*n*=2) and in-hive temperatures (readings from the food chamber, *n*=2) were measured every 5 min during the hottest days (Fig. 4). Peak ambient temperatures reached 45°C inside (Fig. 4A), leading to queen losses (*n*=3). Despite the losses, the remaining mini-colonies were capable of regulating their internal temperature, maintaining a more stable and less variable environment, as demonstrated by the lower standard deviation (s.d.) between the inside (6.31°C) and the outside (10.32°C) temperature, as well as the smaller interquartile range (IQR) inside (11.5°C) compared with the outside (20.1°C). The misting system effectively mitigated some heat stress, activated in 30 min bursts from 12:30 h to 16:00 h, reducing the ambient temperature from 43°C to 30°C (Fig. 4A). Once it stopped, the temperature rose to 40°C.

**Fig. 5.**
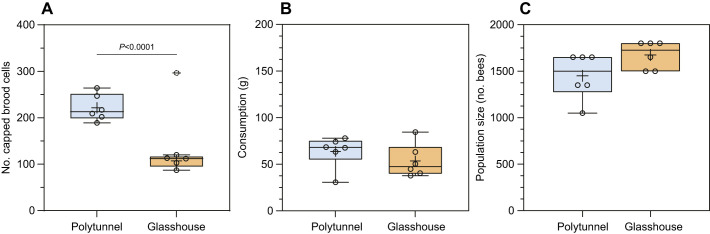
**Semi-field conditions can influence capped brood production in mini-colonies.** Comparison of mini-colony development inside the polytunnel and glasshouse during the first 15 days (one bioassay cycle). Colonies inside the polytunnel (blue; *n*=6) were fed a diet with 15% protein (P15), and those inside the glasshouse (orange; *n*=6) were fed a diet with 18% protein (P18). (A) Mini-colonies in the polytunnel consumed diets with lower protein content compared with those in the glasshouse. The number of capped brood cells in colonies inside the polytunnel was 2 times higher than that in P18 mini-colonies (two-tailed unpaired *t*-test with Welch's correction, *P*<0.05). The dashed circle represents our flagged outlier, which was excluded from our statistical analysis. (B) Mini-colonies inside the polytunnel and the glasshouse showed similar diet consumption (n.s.), ∼60 g during the first 15 days. (C) Mini-colony population size was also similar between the two groups (n.s.), with a mean difference of 225 bees. Box plots show the median (horizontal bar line), upper and lower interquartile range (box, 25th–75th percentiles), with the mean indicated by a plus sign (+); circles represent individual observations.

Different enclosures have different environmental conditions, significantly influencing the mini-colonies’ capacity to rear their brood as seen in Fig. 5A. Here, we excluded one replicate from the P18 diet group: its capped brood had a *z*-score of 2.213, exceeding two standard deviations from the mean, and the mini-colony showed a significant population increase from day 0 to day 15. This met both of our criteria for identifying outliers. Yet, despite similar consumption and population sizes (Fig. 5B,C; unpaired *t*-test, n.s., *P>*0.05), mini-colonies inside the polytunnel were able to rear twice as many capped brood cells as those inside the glasshouse (Fig. 5A; unpaired *t*-test, *P<*0.0001). The main methodological differences between locations were the type of enclosure and the protein content of the diet. As previously discussed, protein content influenced brood-rearing capacity (Fig. 2B,D). And although the P15 diet in the polytunnel was lower in protein, it resulted in 59% more capped cells than in the glasshouse (Fig. 5A), probably because of the glasshouse's higher temperatures (up to 40°C) and poorer ventilation.

### Considerations on temperature control, enclosure design and optimisation

Trials conducted inside the glasshouse proved significantly challenging in keeping temperatures below 37°C. We employed shading nets, misting systems, air conditioning units, pedestal fans and two horizontal industrial extraction fans; however, these measures were insufficient to prevent some queen losses and absconding during heat waves. Additionally, the type of ground floor cover can impact the temperature and air quality within the enclosed space. Ground cover made of concrete slabs has a high thermal mass, leading to the accumulation of bee carcasses, which increases the researcher's susceptibility to heat stress and bee dust-related fever, necessitating regular cleaning. In the purpose-built polytunnel (628 m^3^), the ground was intentionally covered with perennial ryegrass, improving heat absorption and ground sanitation – bee carcasses naturally and quickly decomposed. Flowering was prevented by regular mechanical mowing of any appearance of flower buds. Furthermore, other differences are likely to have affected colony well being. The polytunnel had higher flight activity, better ventilation (netted walls) and a natural floor, unlike the glasshouse's concrete slabs. We recommend using enclosures that more closely mimic natural environments, such as polytunnels, whenever possible.

Other observations made inside the enclosures were foragers’ ability to collect sugar syrup from various feeder types and deliver it to their colony. Feeders providing ample drinking space were emptied within hours, causing some mini-colonies to fill rapidly. When we supplied high-concentration syrup (>50% carbohydrates), we noticed a reduction in the area available for queens to lay eggs. In these mini-colonies, sugar storage can rapidly occur in the middle frames, limiting the number of empty cells available for egg laying and potential brood rearing. A syrup concentration of 33% carbohydrates, similar to the nectar found in wildflowers (Kim et al., 2011), was preferred. However, when artificial flowers ran out of nectar, foragers attempted to expand their foraging range, with many repeatedly contacting the enclosure walls, which probably increased exhaustion and mortality. During this period, light diffusion through the enclosure caused some foragers to become trapped against the wall’s brightest areas, effectively forming ‘light traps’. Restricted foraging may alter colony dynamics; however, the enclosed system was designed to reduce environmental variability and allow for a focused test of nutritional effects on brood. This trade-off highlights the balance researchers must strike between experimental control and ecological realism when studying colony-level nutritional responses.

Future studies should explore methods to mitigate this behaviour by keeping foragers engaged in nectar foraging activities, improving enclosure design through enhanced light dispersion or shading, and implementing more effective cooling systems to mitigate heat shocks during the summer.

### Development and optimisation of a minimally disruptive image-capturing system for standardising honey bee nutritional studies

The need to shake colony frames to release adult bees for image capture, combined with sudden fluctuations in comb temperature, can result in high stress levels for both adults and brood, negatively impacting colony health. The novel device, specifically designed for this bioassay method, sits on top of the mini-colony, allowing us to optimise image timing and minimise disruptions to our mini-colonies during the 15 day comprehensive assessment cycle. Frames with adult bees are shaken sequentially into the inside of the acrylic box, guaranteeing that all nurse bees are placed back in their hive with frames to support them. The acrylic box is made with off-the-shelf anti-glare Perspex acrylic with UV protection, similar to what museums use to protect picture frames. This provides some UV protection to honey bee eggs and larvae, which are sensitive to UV irradiation (Beard, 1972).

The image capture speed on both sides of the frame is approximately ∼1.19 min per frame. A single experienced researcher can complete a full assessment of each mini-colony in ∼6.35 min, i.e. less than 10 min per mini-colony. Previously, two researchers were needed to speed up the process and reduce colony disruption. To the best of our knowledge, this is the quickest and least disruptive method to capture images across all honey bee colony frames. The image size is approximately 2.2 MB, and its quality is assessed by visually confirming it on the touchscreen display. We repeated image capture if images turned out to be out of focus, stray bees covered the combs, or environmental light impeded a clear image (Fig. S2). We captured 800 frame images in one trial, and 39 of them were out of focus or poor quality. Counting cells with eggs and larvae remains challenging, as the device only captures the full depth of the comb located at the centre of the mini-colony frame, about 100 cells per frame side. This is due to the natural individual cell shape that has a hexagonal structure tilted upwards at 9–14 deg and with a depth of approximately 12 mm. In contrast, capped brood and honey cells are easily quantifiable by visually counting each comb cell surface.

The double Raspberry Pi set-up was required because the Raspberry Pi 4 cannot reliably and simultaneously control the Arducam 64MP Hawkeye cameras and the touchscreen display. Therefore, the two cameras were connected to one Raspberry Pi (RPi), the touchscreen display was connected to the second RPi, and an Ethernet cable connected both RPi. However, improvements are still possible in further iterations to increase capture speed and image quality. For example, installing additional cameras would allow us to capture comb edges more effectively, or a scanning method (with a single camera) would enable us to examine each frame from top to bottom. Additionally, making the system completely wireless provides higher flexibility and manoeuvrability in field-type settings. Future enhancements could also incorporate artificial intelligence to capture honey bee larvae more effectively and include small temperature sensors in the acrylic box to alert the researcher if local micro-temperatures that harm the brood are extremely low or high. We hope this method establishes a foundation for standardising future nutritional studies as a whole.

## Supplementary Material

10.1242/jexbio.251151_sup1 Supplementary information
